# Investigation of the Volatile Fraction of Rosemary Infusion Extracts

**DOI:** 10.3797/scipharm.1004-23

**Published:** 2010-06-16

**Authors:** Christine Tschiggerl, Franz Bucar

**Affiliations:** Institute of Pharmaceutical Sciences, University of Graz, Universitätsplatz 4/1, 8010, Graz, Austria

**Keywords:** *Rosmarinus officinalis*, Infusion, GC-MS, Essential oil, SPE

## Abstract

The relative proportions of chemical classes (hydrocarbons, oxides, alcohols, ketones, esters) in the essential oil of rosemary *(Rosmarinus officinalis* L., Lamicaeae) and in the volatile fraction of the infusion extracts were examined and showed remarkable differences.

The volatile compounds of the infusion were isolated by two different methods, hydrodistillation and solid phase extraction (SPE). The main constituents of the volatile fraction of the infusion were (hydrodistillation/SPE): 1,8-cineole (42.4%/44.7%), camphor (31.4%/31.8%), α-terpineol (8.6%/8.1%) and borneol (8.3%/7.8%). The qualitative and quantitative composition of the volatile compounds of the infusion was compared to the essential oil isolated by hydrodistillation directly from the leaves. The major constituents of the essential oil of the leaves were 1,8-cineole (41.6%), camphor (17.0%), α-pinene (9.9%), α-terpineol (4.9%) and borneol (4.8%). Comparison of the total essential oil yield quantified by hydrodistillation of the infusion (0.36% v/w) with the essential oil yield of the leaves (1.84% v/w) revealed that only 19.6% of the initial oil could be extracted by infusion.

## Introduction

Many herbs with potentially beneficial effects which are attributed to the volatile constituents are used as herbal teas, for instance peppermint, lemon balm, thyme or rosemary. In traditional medicine, *Rosmarinus officinalis* L. (Lamiaceae) leaves are used as an integrant of various tea mixtures (up to 350mg/g tea) in cardiovascular, sleep and neuritic disorders [1]. Rosemary has also gained much interest during the last years due to its role as antioxidant in food [2–4]. The most characteristic constituents of the leaves are essential oil, phenolic diterpenes, rosmarinic acid derivatives, flavonoids, triterpenes and steroids [1, 5]. The essential oil composition was subject to various previous studies [6–8]. According to these data the following compounds are found as the main constituents in *R. officinalis* essential oil (REO), considering the fact that compounds vary in chemotype and origin: 1,8-cineole, camphor, α-pinene, camphene, borneol, bornyl acetate, myrcene, limonene, α-terpineol and caryophyllene [1, 5, 9]. Antimicrobial activity, antitumor activity, antispasmodic and anticonvulsant activities and hyperglycemic effects were recognized as pharmacological actions of REO [10, 11].

Antibacterial effects of REO have been reported against different gram positive and gram negative strains [12]. The antimicrobial effects of REO against *Staphylococcus aureus*, *Pseudomonas aeroginosa* and *Candida albicans* were mainly attributed to 1,8-cineole, whereas the effect against *Escherichia coli* was related to monoterpene hydrocarbons [13]. Apart from this, REO shows antioxidative activity which seems to be higher or comparable to that of α-tocopherol [12]. It was reported that these effects are the synergistic result of the oil composition [14]. The antioxidant activity of REO has been compared with that of *Thymus vulgaris* and has been shown to be almost as efficient [15].

*R. officinalis* leaves infusion is widely used in folk medicine as well as for food purposes, however its aromatic composition is only insufficiently investigated [16], a fact which is also true for many other herbal teas.

Therefore, the aim of this study was to compare the essential oil from rosemary infusion extract isolated by two different methods (hydrodistillation versus SPE) and to determine the qualitative and quantitative composition of the volatile compounds from infusion versus original rosemary essential oil (REO).

## Results and Discussion

The composition of the essential oil of *R. officinalis* and that of the volatile fraction of the rosemary infusion obtained by hydrodistillation as well as by SPE were compared.

The qualitative and quantitative results of REO which were in conformity with data from literature are indicated in Table 1 [6–8]. The essential oil yield was 1.84% ± 0.036 (v/w) and its composition was established by 97.4% of its total peak area from the GC-MS chromatograms. Thirty-eight compounds were identified. The main constituents of the essential oil were 1,8-cineole, camphor, α-pinene, α-terpineol and borneol. The other constituents were present in levels of less than 4%.

The aromatic composition of the infusion extracts were established by 98.4% (hydrodistillation) and by 98.9% (SPE) of total peak area from the GC-MS chromatograms. More than 90% of the volatile fraction of the infusion extracts was formed by its main constituents 1,8-cineole, camphor, α-terpineol and borneol. All other constituents were present in levels of less than 3%. Detailed data and standard deviations are shown in Table 1.

Furthermore, the percentages of hydrocarbons, oxides, alcohols, ketones and esters were calculated (Table 1). Oxides were mainly represented by 1,8-cineole, more than 70 % of the oil consisted of oxygen containing compounds.

When we compared the chromatograms of the infusion extracts obtained by hydrodistillation and SPE using qualitative and semiquantitative methods, we found no major differences. However, we noticed significant differences when we focused on the relative proportions of chemical classes of the constituents determined in REO in comparison with both infusion extracts.

In the infusion extracts the amount of hydrocarbons decreased whereas the total level of alcohols and ketones increased. The amount of esters appeared to be inconsiderable in REO and in the infusion extracts. The ratio of oxides in relation to other compound classes remained on a comparable level in both, infusion and REO. The proportion of oxygen containing compounds increased from 74.12 % (REO) to 97.72 % (infusion, hydro-distillation) and 98.42 % (infusion, SPE), respectively, based on % of total peak area.

A plausible explanation by the means of octanol-water partition coefficient and of the boiling points of the major compounds could be attempted.

The significant loss of monoterpene hydrocarbons present in REO can be explained by their higher volatility and lower water solubility (boiling points at about 160°C and log P values of about 4.4–4.6 for α-pinene, camphene, β-pinene, myrcene, *p*-cymene, γ-terpinene [17]. The loss of the sesquiterpene hydrocarbon *trans*-caryophyllene (boiling point at 262°C and log P value of 6.777, at 25°C [17]), present in REO, can only be explained by its lipophility. The higher hydrophility and the less volatility of alcohols and ketones might also explain the fact that although the proportion of oxygen containing compounds significantly increased in the infusion extracts, the ratio oxides vs. alcohol + ketones shifted from 1.35 (REO) to 0.77 (infusion, hydrodistillation) and 0.84 (infusion, SPE), respectively (mean bp of alcohols and ketones > 205°C). According to log P values, hydrophility of 1,8-cineole, α-terpineol and borneol (the latter two represent the major alcoholic compounds in REO) are comparable (2.821, 2.790 and 2.707, respectively, at 25°C [17]), camphor, the main ketone also shows a similar log P value of 2.128 (at 25°C) [17]. A study concerning lemon verbena tea showed an increase of aldehyds and esters and decreasing amounts of hydrocarbons, oxides and alcohols [18].

The enrichment of camphor compared to 1,8-cineole (4:3 in infusions, 5:2 in REO) was even more pronounced in a study where the ratio of the two major compounds, 1,8-cineole and camphor, of 9:1 (infusion) and 5:2 (REO) was found, respectively [16]. Certainly, the relations vary according to chemotype and origin of the plant material. The two compounds 1,8-cineole and camphor amounted up to 70% of the whole essential oil present in the infusion as it also was noticed in the same study, where dissolution rate and kinetics of these two main compounds were studied precisely, but all the other constituents were ignored.

However, some minor compounds which were found in the essential oil obtained by hydrodistillation from the infusion were not detected in the chromatogram of the SPE extract and vice versa, see Table 1. It is obvious that through infusion these highly volatile compounds were lost. Many of the monoterpene hydrocarbons as tricyclene, myrcene, α-phellandrene, α-terpinene, *p*-cymene, ocimene, terpinolene and the sesquiterpene hydrocarbons *trans*-caryophyllene and α-humulene occurring in REO were not detected in the infusion. This loss can be explained by the tea preparation procedure with boiling water and was also observed in a study concerning *Salvia officinalis* infusion [19].

Recovery rates and results of the quantitative analysis of the volatile fraction obtained by SPE procedure are shown in Table 2. Mean values of the individual compounds and SDs (n=5) were calculated. Calibration data of the compounds (Table 2) indicated linearity of the detector signal within the concentration range injected (R^2^ ≥ 0.9956).

Comparison of the total essential oil yield quantified by hydrodistillation of the infusion 0.36% ± 0.040 (v/w), with the essential oil content of the leaves 1.84% ± 0.036 (v/w), obtained by Clevenger apparatus, revealed that only 19.6% of the initial oil could be extracted by hydrodistillation of the infusion. Similarly, a low portion of volatiles was noticed in a study where Roman chamomile tea was investigated [20]. The original infusion after 3 hours hydrodistillation was also verified if there were traces of volatiles by doing an SPE extraction and GC-MS analysis. We found only traces of the major compounds around the detection limit. A total essential oil content of 4.26–4.38 mg/100ml infusion could be calculated based on an average relative density of 0.895–0.920 for REO [21]. Compared to the recommended daily dose of 10–20 drops of essential oil, which corresponds to 190– 380 mg, the recommended daily two or three cups of rosemary tea only contain a small proportion (6.39–6.57 mg per cup) of these active volatiles [1, 5, 22]. Similar amounts of essential oil, 1.3 to 11.4 mg per cup of tea, where found in different fennel teas [23].

As a conclusion, SPE seems to be an elegant alternative to hydrodistillation and a comparable but more economic method for determining the aromatic composition of infusion extracts. The volatility of the individual compounds has a significant influence on their extractability during preparation of an infusion and significant losses of volatiles, above all hydrocarbons, can be expected. The fact that the volatile fraction of rosemary tea differs considerably compared to the original rosemary essential oil seems to be pharmaceutically relevant.

## Experimental

### Plant Material

A commercial sample *R. officinalis* leaves was obtained from Mag. Kottas, Vienna (Austria). The material complied with the monograph of the European Pharmacopoeia [21]. A voucher specimen is kept at the Department of Pharmacognosy, Institute of Pharmaceutical Sciences, University of Graz.

### Chemicals

All reagents and solvents used were of analytical or HPLC grade. Solvents and materials were purchased from the following suppliers: n-hexane (Fluka, Switzerland), methanol (Merck, Germany), methylene chloride (Roth, Germany), sodium sulfate (Roth, Germany). Authentic standards were 1,8-cineole (Fluka, Switzerland), (1*R*)-(+)-campher (Aldrich, Germany), linalyl acetate (Fluka, Switzerland), (+)-borneol (Fluka, Switzerland), (−)-verbenone (Fluka, Switzerland), (+)-α-terpineol (Fluka, Switzerland), (+/−)-linalool (Roth, Germany). The n-alkanes C_8_–C_26_ for the determination of the linear retention index were from Sigma, USA. Isolute C18 (EC) columns (1g, 15ml) from Biotage, Sweden were used as SPE cartridges.

### Gas chromatography – mass spectrometry

The composition of the essential oil and of the volatile compounds of the infusion extracts was determined by GC/MS. Each sample was analyzed ternary. Analyses were performed using an Agilent 7890A GC system coupled with an Agilent 5975C MSD operating at 70 eV, ion source temperature 230°C, interface temperature 280°C. A split injection (split ratio, 80:1) at 240°C injector temperature was utilized. Injection volumes were 1μl. A fused silica capillary column 5% phenylmethylsiloxane (HP-5MS 30 m x 250 μm x 0,25 μm, Agilent J & W, USA) was used. The temperature program was as follows: 2 min at 45°C, then to 250°C at 4°C/min, finally held at 250°C for 2 min. The carrier gas was helium 5.6 at a flow rate 0.9ml/min. Data acquisition was performed with Agilent GC/MSD ChemStation Version E.02.00 for the mass scan range 40–300u.

Compounds were identified by retention indices [24] and by comparing their mass spectra with spectral data libraries [24, 25] and a laboratory own data base. Furthermore, for some compounds pure standard substances were available.

### Essential oil hydrodistillation

Hydrodistillation procedures were done according to the European Pharmacopoeia [21]. 25g of rosemary leaves were hydrodistilled for 3 hours. The infusion (4500ml) was also hydrodistilled for 3 hours immediately after preperation to avoid loss of volatiles. Five separate analyses were performed of each experiment. The essential oil samples were dried over anhydrous Na_2_SO_4_ and stored in dark glass bottles at −20°C until analysis. The oil samples were diluted with hexane (1:30) before GC/MS analysis.

### Preparation of rosemary infusion

An infusion was prepared according to literature [1]. Boiling distilled water (4500 ml) was poured onto rosemary leaves (60 g), and the infusion was left to brew for 15 min. Then it was filtered and rinsed three times with distilled water and brought to a final volume of exactly 4500ml. These high amounts of infusion were necessary to determine the quantitative essential oil content with the Clevenger apparatus. The same tea preparation procedure with 450 ml boiling water and 6 g leaves was done before SPE extraction. Each experiment was performed fivefold.

### Solid phase extraction (SPE) of the infusion

The following method was adapted from literature [19]. An Isolute C18 (EC) solid phase extraction cartridge (1 g) was conditioned twice with 8 ml methylene chloride and twice with 8 ml methanol. Afterwards 8 ml of distilled water was passed through the cartridge twice and not allowed to dry before the filtered infusion was applicated. The infusion was loaded onto the cartridge with a flow of 1–2 ml/min. The cartridge was dried for 15 min by putting on a slight vacuum. The compounds retained on the SPE column were eluted into a 5 ml graduated flask with exactly 5 ml of methylene chloride, which contained the internal standard, linalyl acetate (200 ng/μl). Pure dichloromethane was added to a final volume of 5 ml. Five separate determinations were performed. These samples were stored in glass bottles at −20°C until they were used for GC/MS analysis.

### Semiquantitative analysis

REO and the essential oil from the infusion obtained by hydrodistillation were quantified by the area% method without considering calibration factors. For comparison, the SPE extract from the infusion was quantified with the same method, without considering the internal standard.

### Quantitative analysis

Quantification and determination of recovery rates using the method of internal standard were done for the major compounds in the extract obtained by SPE. Standard solutions were prepared containing 50, 250, 500, 750 and 1000 ng/µl borneol, verbenone, camphor, α-terpineole, linalool and the same quantity (200 ng/µl) of the internal standard linalyl acetate, a substance which was absent in REO and also in the infusion extracts and which peak did not interfere with other substances in the chromatograms.

Four 1,8-cineole standard solutions were prepared containing 100, 300, 600, 900, 1200 ng/μl and the same quantity of the internal standard (200 ng/μl). Within this range of concentrations the detector response was linear. The compounds for the standard solutions were dissolved in hexane. These standard solutions were used for creating calibration curves by linear regression (peak area compound / peak area internal standard versus concentration compound / concentration internal standard).

Quantity of each major constituent was calculated by using the following formula:

$${\text{m}}_{\text{i}}=\frac{({\text{amount}}_{\text{Istd}})\times ({\text{area}}_{\text{i}})\times {\text{rf}}_{\text{i}}}{{\text{area}}_{\text{Istd}}}$$

where *m_i_* is the total amount of substance i in the sample (ng), *amount_Istd_* the amount of internal standard which was added to the sample (ng), *area_i_* the peak area of the substance i, *rf_i_* the response factor obtained from the slope of the calibration curve and *area_Istd_* the peak area of the internal standard [26].

Determination of the recovery rates for the major compounds found in the SPE extract were done by adapting the SPE procedure according to section 2.6. Instead of the original infusion an essential oil free infusion, obtained by boiling for 2 hours to eliminate the volatiles, was used. Elution was done with 5 ml of methylene chloride, which contained the internal standard linalyl acetate (200 ng/µl) and the same amount of borneol, 1,8-cineole, camphor, α-terpineol, verbenone and linalool. Calculation was done by using the following formula :

$$\text{RR}=\frac{{(\text{area}}_{i})\times {(\text{amount}}_{\text{Istd}})\times 100\times {\text{rf}}_{i}}{\left({\text{area}}_{\text{Istd}})\times ({\text{amount}}_{\text{i}}\right)}$$

*RR* is the recovery rate of substance i in percent, for other abbreviations see above.

## Figures and Tables

**Tab. 1.** Percentage composition^a^ of the essential oil of *Rosmarinus officinalis* and of the volatile fraction of the infusion extracts

**Compound/Chemical class**	**RI^b^**	**Percentage from total area**			**Ident^c^**
		**REO (n=5)**	**Infusion extracts (n=5)**		
			**Hydrodistillation**	**SPE (C18)**	

Tricyclene	920	0.10 (0.004)	n.d.	n.d.	1,2
α-Pinene	932	9.92 (0.358)	0.16 (0.051)	n.d.	1,2
Camphene	946	3.22 (0.113)	0.11 (0.025)	n.d.	1,2
β-Pinene	974	3.18 (0.079)	0.10 (0.021)	n.d.	1,2
Myrcene	991	1.32 (0.030)	n.d.	n.d.	1,2
α-Phellandrene	1003	0.24 (0.006)	n.d.	n.d.	1,2
α-Terpinene	1015	0.71 (0.015)	n.d.	n.d.	1,2
*p*-Cymene	1025	1.90 (0.070)	0.17 (0.018)	n.d.	1,2
β-(*Z*)-Ocimene	1038	0.09 (0.006)	n.d.	n.d.	1,2
γ-Terpinene	1058	0.75 (0.016)	0.13 (0.017)	n.d.	1,2
*cis*-Sabinene hydrate	1066	0.12 (0.007)	n.d.	0.25 (0.011)	1,2
Terpinolene	1087	0.40 (0.008)	n.d.	n.d.	1,2
*trans*-Sabinene hydrate	1097	0.10 (0.007)	n.d.	0.23 (0.012)	1,2
*trans*-Caryophyllene	1419	1.02 (0.065)	n.d.	n.d.	1,2
α-Humulene	1453	0.23 (0.016)	n.d.	n.d.	1,2
**Total Hydrocarbons**		**23.30 (0.513)**	**0.67 (0.127)**	**0.48 (0.021)**	

1,8-Cineole	1033	41.58 (0.493)	42.43 (1.254)	44.65 (1.043)	1,2,3
Caryophyllene oxide	1584	0.52 (0.042)	n.d.	n.d.	1,2
**Total Oxides**		**42.10 (0.469)**	**42.43 (1.254)**	**44.65 (1.043)**	

1-Octen-3-ol	980	0.22 (0.007)	0.49 (0.033)	0.25 (0.024)	1,2
Linalool	1100	1.30 (0.034)	2.08 (0.094)	1.52 (0.083)	1,2,3
*endo*-Fenchol	1113	0.08 (0.005)	0.09 (0.004)	n.d.	1,2
Isopulegol	1155	0.10 (0.010)	0.13 (0.014)	n.d.	1,2
Borneol	1165	4.84 (0.157)	8.34 (0.306)	7.79 (0.297)	1,2,3
Terpinen-4-ol	1176	1.10 (0.036)	1.93 (0.075)	1.44 (0.074)	1,2
*p*-Cymen-8-ol	1185	0.10 (0.003)	0.13 (0.007)	0.16 (0.017)	1,2
α-Terpineol	1191	4.85 (0.180)	8.62 (0.352)	8.05 (0.446)	1,2,3
Myrtenol	1195	0.14 (0.004)	0.20 (0.006)	0.22 (0.022)	1,2
Thymol	1294	0.29 (0.020)	0.34 (0.041)	0.33 (0.035)	1,2,3
Carvacrol	1303	0.11 (0.008)	0.13 (0.011)	n.d.	1,2
Caryophylladienol I	1637	0.19 (0.017)	n.d.	n.d.	1,2
**Total Alcohols**		**13.32 (0.437)**	**22.48 (0.862)**	**19.76 (0.918)**	

3-Octanone	986	0.11 (0.005)	0.16 (0.014)	n.d.	1,2
Camphor	1145	16.97 (0.283)	31.39 (0.252)	31.82 (0.659)	1,2,3
*trans*-Pinocamphone	1159	0.10 (0.002)	0.14 (0.007)	n.d.	1,2
Pinocarvone	1161	0.11 (0.003)	0.17 (0.006)	0.11 (0.034)	1,2
*cis*-Pinocamphone	1173	0.13 (0.003)	0.19 (0.006)	0.16 (0.011)	1,2
Verbenone	1208	0.45 (0.014)	0.42 (0.023)	1.29 (0.119)	1,2,3
**Total Ketones**		**17.87 (0.300)**	**32.47 (0.276)**	**33.38 (0.618)**	

Bornyl acetate	1287	0.62 (0.025)	0.34 (0.029)	0.20 (0.013)	1,2
Humulene epoxide II	1610	0.09 (0.008)	n.d.	n.d.	1,2
(*Z*)-Methyljasmonate	1649	0.12 (0.008)	n.d.	0.43 (0.062)	1,2
**Total Esters**		**0.83 (0.037)**	**0.34 (0.029)**	**0.63 (0.070)**	

Unknown	1202	0.07 (0.002)	0.10 (0.005)	n.d.	1,2
Unknown	2427	n.d.	n.d.	0.55 (0.120)	
Unknown	2441	n.d.	n.d.	0.27 (0.103)	

**∑ Peak area %**		**97.49**	**98.49**	**99.72**	

a Calculated by the peak area % method without consideration of calibration factors;

b linear retention index relative to C_8_–C_26_ n-alkanes on a HP5-MS column;

c Mode of Identification: 1=mass spectral libraries, 2=retention index, 3= co-chromatography with reference compound; n.d. …not detected; tr…trace; SDs are given in parentheses.

**Tab. 2.** Quantification of the major volatile compounds of rosemary leaves infusion and calibration data*

**Compound name**	**Regression equation**	**R^2^**	**Recovery rate% (n=3)**	**mg/100ml infusion^a^ (n=5)**
1,8-Cineole	y = 0.6347x + 0.1294	0.9967	75.2 (6.14)	1.772 (0.2391)
Camphor	y = 0.5888x + 0.0953	0.9958	70.9 (7.00)	1.241 (0.1518)
α-Terpineol	y = 0.6922x + 0.0926	0.9978	60.8 (3.03)	0.430 (0.0485)
Borneol	y = 0.7787x + 0.0397	0.9956	81.3 (3.32)	0.350 (0.0351)
Terpinen-4-ol	y = 0.6922x + 0.0926	0.9978	60.8 (3.03)	0.077 (0.0082)
Linalool	y = 0.6899x + 0.1106	0.9975	70.6 (3.97)	0.069 (0.0062)
Verbenone	y = 0.9101x + 0.0999	0.9989	88.4 (5.06)	0.062 (0.0056)
∑				4.001 (0.4816)

* Compounds were recovered by SPE and quantified using internal standard method; y…concentration compound / concentration internal standard (ng/μl); x…peak area compound / peak area internal standard; R^2^…correlation coefficient;

a respecting recovery rates; SDs are given in parentheses.
